# Microdiversity sustains the distribution of rhizosphere-associated bacterial species from the root surface to the bulk soil region in maize crop fields

**DOI:** 10.3389/fpls.2023.1266218

**Published:** 2023-10-12

**Authors:** Xianheng Fu, Qi Fu, Xiaozheng Zhu, Xian Yang, Huaihai Chen, Shiqing Li

**Affiliations:** ^1^ State Key Laboratory of Biocontrol, School of Ecology, Shenzhen Campus of Sun Yat-sen University, Shenzhen, Guangdong, China; ^2^ State Key Laboratory of Soil Erosion and Dryland Farming on the Loess Plateau, Institute of Soil and Water Conservation, Chinese Academy of Sciences and Ministry of Water Resource, Shaanxi, China

**Keywords:** bacteria, fungi, persistence, variability, maize rhizosphere

## Abstract

Over the years, the microbial community of maize (*Zea mays*) rhizosphere has been extensively studied; however, the role of microdiversity sustain rhizosphere-associated microbial species distribution from root surface to bulk soil in mature maize is still unclear. Although operational taxonomic units (OTUs) have been used to classify species, amplicon sequence variants (ASVs) have been shown to be effective in representing microdiversity within OTUs at a finer genetic scale. Therefore, the aim of this study was to examine the role of microdiversity in influencing the distribution of rhizosphere-associated microbial species across environmental gradients from root surface to bulk soil at the OTU and ASV levels. Here, the microbial community structures of bulk, loosely bound, and tightly bound soil samples from maize rhizosphere were examined at OTU and ASV levels. The results showed that OTU and ASV methods exhibited similar microbial community structures in rhizosphere. Additionally, different ecotypes with varying distributions and habitat preferences were observed within the same bacterial OTU at the ASV level, indicating a rich bacterial microdiversity. In contrast, the fungal community exhibited low microdiversity, with no significant relationship between fungal microdiversity and persistence and variability. Moreover, the ecotypes observed within the bacterial OTUs were found to be positively or negatively associated with environmental factors, such as soil organic carbon (SOC), NO_3_^−^–N, NH_4_^+^–N contents, and pH. Overall, the results showed that the rich microdiversity could sustain the distribution of rhizosphere-associated bacterial species across environmental gradients from root surface to bulk soil. Further genetic analyses of rhizosphere-associated bacterial species could have considerable implications for potential mediation of microdiversity for sustainable crop production.

## 1 Introduction

The rhizosphere is a biological hotspot that serves as an interaction site between plant roots, microorganisms, and soil constituents (Kuzyakov et al., 2003; Kuzyakov and Blagodatskaya, 2015; Fan et al., 2017). Plant roots release various exudates, such as low-molecular weight organic acids and metabolites, and take up water and nutrients, which influences soil pH, nutrition levels, and microbial community composition, called rhizosphere effect (Philippot et al., 2013; Kuzyakov and Blagodatskaya, 2015; Kuzyakov and Razavi, 2019). Research on the genetic structure of the soil microbiome of plant rhizosphere could improve the understanding of plant driving microbial community distribution patterns in rhizospheric soil (Smith et al., 1995; Di Cello et al., 1997; Baraniya et al., 2016). The rhizosphere microbial community of maize (*Zea mays*) has been extensively studied because it is an economically important crop across the globe (Gore et al., 2009; Peiffer et al., 2013; Vescio et al., 2021). Previous studies reported that cultivar, genotype, and growth stage of maize, and soil basic properties, especially mineral nutrition, can affect the microbial community composition of maize rhizosphere (Kandeler et al., 2002; Schmalenberger and Tebbe, 2002; Castellanos et al., 2009; Aira et al., 2010). Since the dynamics of the bacterial community structures in the maize rhizosphere during different growth stage have been reported (Li et al., 2014), it has been found that the distance decay relationship (from the root surface to bulk soil) can reflect variations in bacterial community diversity associated with soil properties (Fan et al., 2017). For example, two major inorganic nitrogen forms (NH_4_^+^ and NO_3_^-^) available for plant growth can affect soil bacterial communities with increasing distance from root surface of maize (Zhang et al., 2021). Similarly, the maize rhizosphere exhibits a reduced microbial α-diversity compared to the bulk soil as a result of the rhizosphere effect (Kandeler et al., 2002; Peiffer et al., 2013). Notably, within a 16S rRNA gene-defined taxon (fine genetic scale) encompasses distinct ecotypes or lineages that respond differently to environmental variation over space and time (Hunt et al., 2008; Martiny et al., 2009; Eren et al., 2015). However, at fine genetic scale, microbes respond to variation from root surface to bulk soil of mature maize plants growing field conditions still remain poorly understood.

Operational taxonomic units (OTUs) are commonly used in assessing microbial community diversity and abundance in the environment (Benlloch et al., 1995; Thompson et al., 2017). Generally, OTUs are defined as 97% sequence similarity of the target barcode region (16S for bacteria and ITS for fungi), determined using an agglomerative clustering algorithm (Weisburg et al., 1991; Schoch et al., 2012; Tipton et al., 2021). OTUs have been linked to the equivalence of microbial species or species hypotheses (Stackebrandt and Goebel, 1994; Tipton et al., 2021). While this classification of microbial diversity can capture broad taxonomic shifts, it provides limited genetic resolution at this loosely defined species level (Chase Alexander et al., 2017). Growing evidence indicates that ecological heterogeneity within OTUs is caused by phylogenetic inconsistency and not the ‘lumping’ of taxa resulting from lower identity cut-offs (Jaspers and Overmann, 2004; Koeppel and Wu, 2013; Larkin et al., 2016). Thus, microbial sub-taxa within larger phylogenetic groups that have distinct niche space and greater than 97% 16S rRNA gene similarity is referred to as microdiversity (Larkin and Martiny, 2017). Recently, amplicon sequence variants (ASVs) have been proposed as an alternative to OTUs for analysing microbial community diversity at a finer genetic scale (Callahan et al., 2017; Chiarello et al., 2022). The genetic resolution provided by ASVs is desirable given the amount of variability within larger phylogenetic groups (i.e., OTUs) (Martiny et al., 2013; Tipton et al., 2021). Compared with OTUs, ASVs unveiling previously overlooked ecological patterns in different ecosystems, including soil (Chase Alexander et al., 2017; Scales et al., 2022), lake (Herren et al., 2016; García-García et al., 2019), and oceans (Chafee et al., 2018). Microdiversity has been detected in the maize rhizosphere in previous studies. For example, Di Cello et al. (1997) reported significantly higher microdiversity of *Burkholderia cepacia* population in the rhizosphere of maize in the early stages of root growth. Similarly, Dalmastri et al. (1999) compared the effects of soil type, maize cultivar, and root location (i.e., rhizosphere and rhizoplane) on the microdiversity of root-associated *B. cepacia* populations. However, studies are yet to compare the effectiveness of OTUs and ASVs in detecting the microdiversity of root-associated soil compartments of mature maize plants growing under field conditions.

Microbial species consist of many different sub-taxa that sustain their distribution across broad environmental gradients in oceans (Kashtan et al., 2014; Kashtan et al., 2017). However, the extent to which these sub-taxa contribute to the preservation of species distributions along environmental gradients from the root surface to the bulk soil in maize remains largely unexplored. Further genetic analyses are imperative to enhance our understanding of soil bacterial diversity, elucidate the functional role of indigenous microorganisms, and investigate the biological changes associated with environmental perturbations in order to establish the intricate relationships among microbial diversity, soil and plant quality, and agricultural sustainability.

Therefore, the aim of this study was to elucidate the microdiversity of microbial communities and the dynamics of ecotypes across environmental gradients from the root surface to the bulk soil of maize under field conditions. OTUs were compared with ASVs to discern distinct ecotypes within species and investigate their influence on species distribution. We hypothesized that: 1) the microdiversity could sustain species distribution along environmental gradients from the root surface to the bulk soil. 2) The distinct ecotypes are associated with environmental variables of the maize rhizosphere from the root surface to the bulk soil.

## 2 Materials and methods

### 2.1 Site description

The three-year (2019-2021) field experiment was conducted at the Cao Xinzhuang Research Station (34°31′N, 108°100′E, 520 m altitude), Yangling, Shaanxi Province, China. The site is characterized by a temperate monsoonal climate. The average annual precipitation is 660 mm, which mainly occurs from July–September. The annual mean air temperature is 12.9°C. The soil type at the study site is an Earth-cumuli-Orthic Anthrosol, according to the Chinese Soil Taxonomy (Li et al., 2021) and the soil texture is silty clay loam. In the upper 20-cm soil layer, soil organic carbon and total nitrogen contents are 7.97 ± 0.31 g kg^-1^ and 0.83 ± 0.05 g kg^-1^. Soil pH is approximately 8.36 ± 0.03. Soil bulk density is 1.42 ± 0.06 g cm^-3^. Total phosphorus and available phosphorus content are 0.71 ± 0.02 g kg^-1^ and 12.45 ± 0.72 mg·kg^-1^.

### 2.2 Experimental design and soil samples collection

Field experiments were conducted in a summer maize field. Plant spacing was 25 cm, row spacing was 70 cm, and sowing depth was 5 cm within the maize field plots (30 m ×20 m). Maize was sown (June 25 in 2019, June 18 in 2020 and June 27 in 2021) using a hand-powered hole-drilling machine. Soil samples were collected during maize flowering stage (Peiffer et al., 2013) in September 2019, 2020, and 2021. Six maize plants were randomly dug out in the middle of the plot to avoid border effects potentially attributable to increased nutrient availability to the border plants of the plot and to keep the root system as intact as possible (Peiffer et al., 2013). Three soil types were collected: loosely bound soil, soil lightly adhered to the root was gently shaken off; tightly bound soil, soil tightly adhered to the root was collected by brushing (Fan et al., 2017; Fan et al., 2018); and bulk soil, the topsoil (0–15 cm) was sampled 35 cm away from plants (in between rows), using an auger corer (Fan et al., 2017; Fan et al., 2018). Tightly bound soil is regarded as rhizosphere soil based on root proximity (Donn et al., 2015). All soil samples were transported to the laboratory in ice boxes (4°C) and sieved using a 2-mm sieve to remove fine plant debris and stones. Finally, six replicates for each soil were collected: tightly bound, loosely bound, and bulk soil. In September 2021, the samples were divided into two parts: one stored in a refrigerator at –40°C for a week prior to DNA extraction and the other part was used for soil chemical analysis.

### 2.3 Soil physiochemical analysis

Soil pH was determined in a soil-water solution (1:5) using a precalibrated pH electrode. The soil NO_3_^−^–N and NH_4_^+^–N contents were measured using a Seal Auto Analyzer after extraction with 2 M KCl at a 1:5 ratio. The soil was air-dried and sieved through a 1-mm sieve for total nitrogen (TN) content determination. TN content was measured using the Kjeldahl method. Soil organic carbon (SOC) content was analysed using the dichromate oxidation method. Briefly, 0.20 g of air-dried soil was digested with a mixture of 0.8 M K_2_Cr_2_O_7_ and H_2_SO_4_ (each 5 mL) for 5 min at 175°C, and then titrated against 0.5 M FeSO_4_. The physiochemical properties of each soil sample are shown in **Tables S1**.

### 2.4 DNA extraction, bioinformatics and statistical analysis

Each soil sample (0.5 g) was extracted using the FastDNA^®^ Spin Kit for Soil (Qbiogene, Germany), according to the manufacturer’s instructions. The 16S rRNA gene was amplified using primer sets 341F (5′-CCTACGGGAGGCAGCAG-3′) and 806R (5′- GACTACHVGGGTATCTAATCC-3′) (Lee et al., 2012). The ITS gene was amplified using primer sets 1737F (5′-GGAAGTAAAAGTCGTAACAAGG-3′) and 2043R (5′-GCTGCGTTCTTCATCGATGC-3′) (Lu et al., 2013). The 16S rRNA and ITS gene fragments from each soil sample were sequenced using an Illumina HiSeq platform (Illumina HiSeq, Personalbio, Shanghai). The open source software QIIME2 was used for sequence read analysis (https://www.qiime2.org/). Amplicon sequence variants (ASVs) were defined using the divisive amplicon denoising algorithm (DADA2) method (Callahan et al., 2016). Taxonomic assignments were performed using the Silva 16S rRNA (www.arb-silva.de) and UNITE (http://unite.ut.ee) reference databases. A total of 7597 ASVs were obtained from bacteria and 1920 ASVs from fungi. Thereafter, ASVs in the bacteria and fungi were clustered into OTUs with 97% similarity using the Opticlust algorithm (Westcott and Schloss, 2017). A total of 4680 OTUs were obtained from bacteria and 1589 OTUs from fungi after removing sequences that were non-bacterial and non-fungal, respectively. Additionally, the terms “OTU” and “ASV” are used throughout the manuscript for the sake of brevity, although ASVs can also be defined as OTU. Sequences obtained from this research were submitted in the NCBI Sequence Read Archive (SRA) with accession number PRJNA997935.

To ensure comparability, 16S rRNA and ITS sequences were rarefied to the 11000 and 17000 sequencing depth for bacteria and fungi, respectively. Principal coordinate analysis (PCoA) was performed to visualise the microbial community structure of three soil types based on unweighted UniFrac distances. The unweighted UniFrac distance calculated from OTU table and ASV table, respectively. Procrustes analysis was used to compare PCoAs (OTUs vs. ASVs) (Peres-Neto and Jackson, 2001). ANOSIM and ADONIS tests were used to test the cluster significance of samples from the unweighted UniFrac distances, which were calculated using OTU and ASV abundance tables. In addition, to examine the habitat preferences of each ASV (from root surface to bulk soil), we normalised them to z-score, which facilitated the visualisation of habitat preferences of low abundance ASVs.

The effects of microdiversity on the persistence and variability of each OTU were examined. Microdiversity was calculated according to García et al. (2019). Briefly, the microdiversity of each OTU was calculated based on the abundance of its constituent ASVs as the exponential of the Shannon index (Jost, 2006; García-García et al., 2019). This value represents the effective number of ASVs in each OTU. Its calculation is conceptually similar to the calculation of the effective number of species in a community but uses ASVs as units of diversity and restricts the sampling space to each individual OTU (García-García et al., 2019).

To detect the persistence and variability of each OTU. Persistence was defined as the proportion of samples containing the group of interest or fraction of samples in which the taxon had at least one count, and variability was defined as the standard deviation of the OTU divided by the mean abundance of the OTU (Shade and Gilbert, 2015; Herren et al., 2016; Linz et al., 2017; García-García et al., 2019). persistence OTUs were selected as persistence > 0.75 and variability < 2 (García-García et al., 2019). One-way analysis of variance (ANOVA) was performed to determine the statistically significant differences (*p* < 0.05) based on the data that followed a normal distribution. All statistical analyses were performed in R (v4.0.1; http://www.r-project.org/), using the “vegan,” “stats,” “scales,” “recharts,” “dplyr,” “phyloseq,” and “ape” packages.

## 3 Results

### 3.1 Microbiome in maize rhizosphere and bulk soil

**Figure 1:**
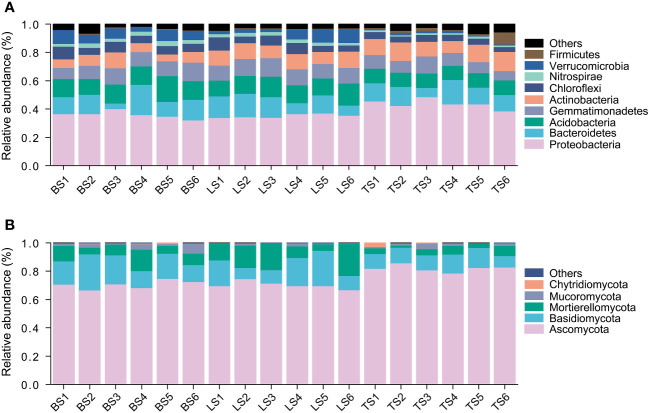
The relative abundance of bacteria **(A)** and fungi **(B)** in root-associated soil compartments of maize. BS, bulk soil; LS, loosely bound soil; TS, tightly bound soil.

In this study, a total of 7597 ASVs were retained from 834,439 high-quality bacterial sequences across the soil samples. Bacterial community mainly consisted of Proteobacteria, Bacteroidetes, Acidobacteria, Gemmatimonadetes, Actinobacteria, Chloroflexi, Nitrospirae, and Verrucomicrobia (**Figure 1A**). ANOVA revealed that Proteobacteria and Bacteroidetes had significantly higher relative abundance in the tightly bound soil than in the loosely bound soil and bulk soil (**Figure 1A**, **Table S2**). Acidobacteria, Chloroflexi, and Verrucomicrobia had significantly higher relative abundance in loosely bound soil and bulk soil than in tightly bound soil (**Figure 1A**, **Table S2**).

Similarly, 1920 ASVs were retained from 443,851 high-quality fungal sequences across the soil samples. Fungal community mainly consisted of Ascomycota, Basidiomycota, and Mortierellomycota (**Figure 1B**). Ascomycota had significantly higher relative abundance in the tightly bound soil than in the loosely bound soil and bulk soil (**Figure 1B**, **Table S3**). Basidiomycota had significantly higher relative abundance in the loosely bound soil and bulk soil than in the tightly bound soil (**Figure 1B**, **Table S3**).

**Figure 2:**
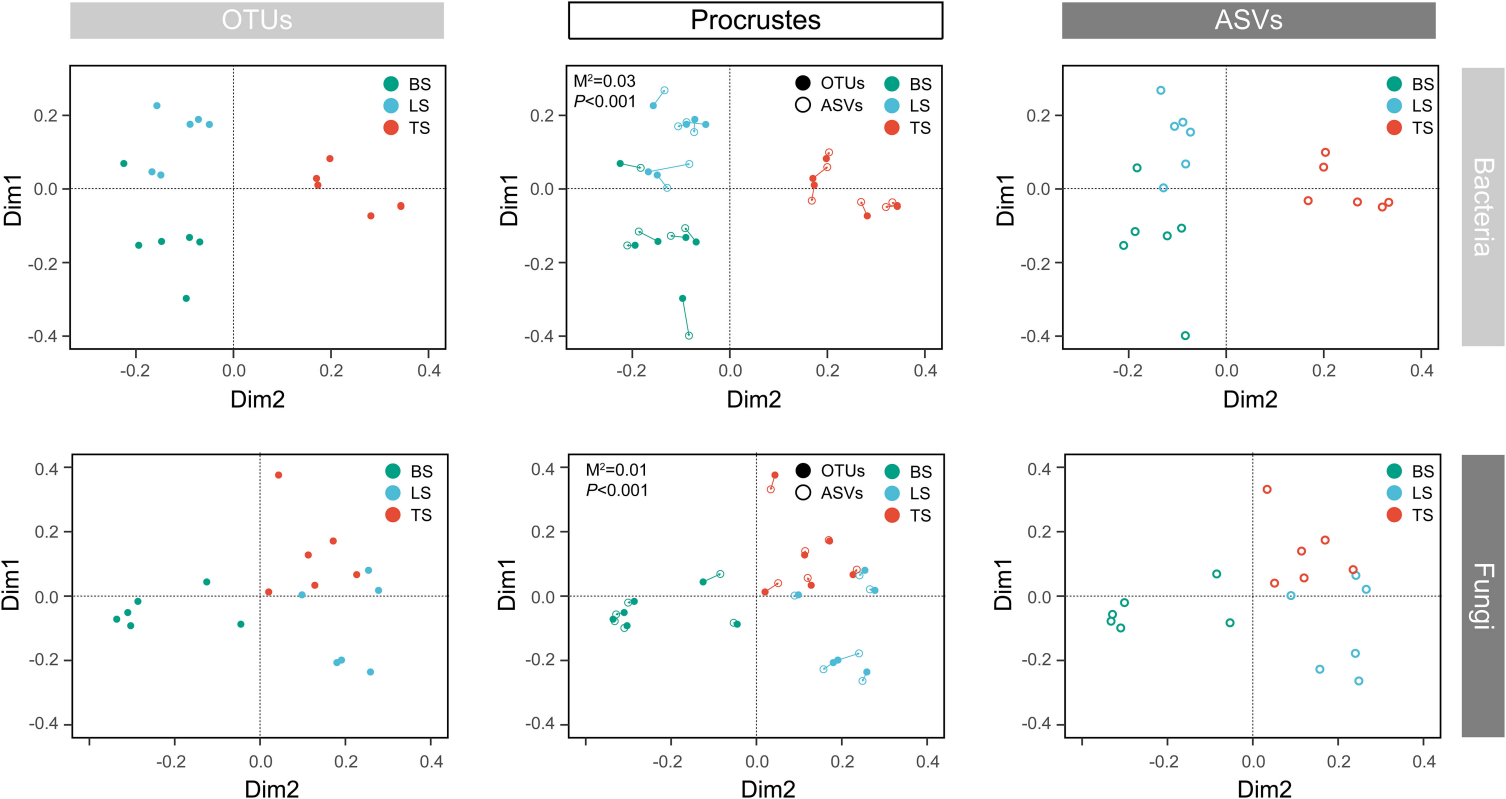
Principal coordinate analysis (PCoA) of the microbial community structure in the rhizosphere of mature maize at the OTU and ASV levels. Procrustes analysis shows the difference between the OTU and ASV levels. The PCoA was based on unweighted UniFrac distances. OTU, operational taxonomic unit; ASV, amplicon sequence variants; PCoA, principal coordinate analysis.

**Figure 3:**
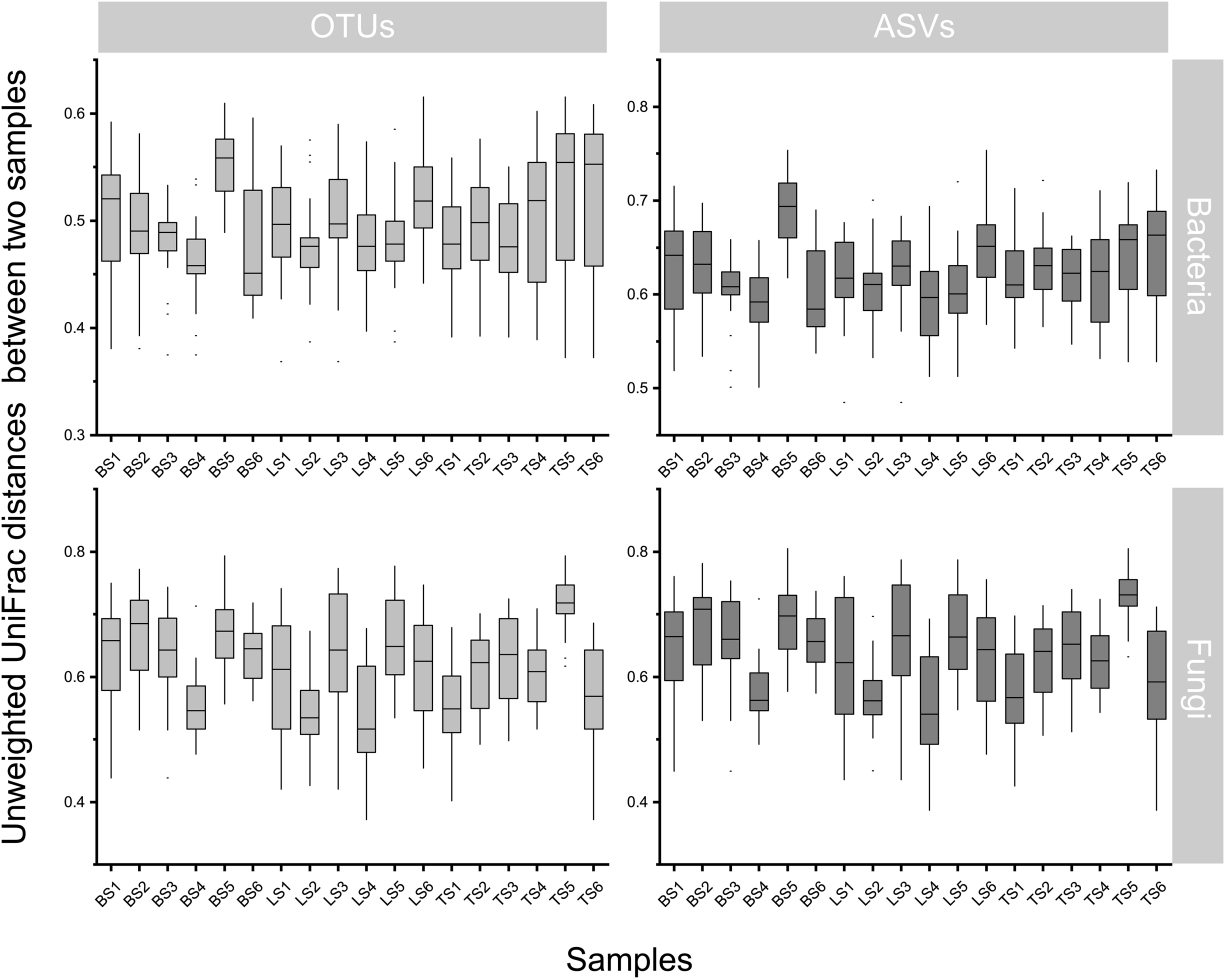
Similarity plots of microbiota in root-associated soil compartments of maize. Similarity in community composition for bulk, loosely bound, and tightly bound soils. Boxplot shows the pairwise unweighted UniFrac distances between soil samples (OTUs vs ASVs, Mantel test, *p* < 0.001, R = 0.975). The x-axis shows each sample. OTU, operational taxonomic unit; ASV, amplicon sequence variants.

To evaluate bacterial and fungal community composition, the principal coordinate analysis (PCoA) of unweighted UniFrac distances was used. At the OTU and ASV levels, PCoA exhibited that the bacterial and fungal communities were aggregated by soil compartments (**Figure 2**). Procrustes analysis showed that the clustering of samples into soil compartments showed similar trends at both OTU and ASV levels under bacterial community (**Figure 2**). ANOSIM and ADONIS tests indicated that both bacterial and fungal community structures in tightly bound soil significantly differed from those in loosely bound and bulk soils (**Table. S4**). Similarly, boxplot of pairwise unweighted UniFrac distances between samples showed that microbial diversity in each sample showed similar trends at both the OTU and ASV levels (Mantel test, *p* < 0.001, R = 0.975, **Figure 3**). The microbial α-diversity showed significant differences across the soil compartments (**Table 1**). The Shannon and Chao1 indices of bacterial community in tightly bound soil were lower than those of bacterial communities in loosely bound and bulk soils. Similarly, the Shannon and Chao1 indices of fungi in tightly bound soil were lower than those of fungi in loosely bound or bulk soils. Moreover, α-diversity was significantly correlated with environmental variables for bacteria and fungi (**Tables S5, S6**).

**Table 1:** Comparison of α-diversity indexes among three soil compartments.

Microbes	α-diversity	Compartments		
		BS	LS	TS
Bacteria	PD	71.41 ± 9.67a	56.82 ± 4.62b	54.88 ± 1.67b
	Pielou_eveness	0.93 ± 0.01a	0.92 ± 0.01a	0.91 ± 0.01b
	Chao1	1071 ± 258a	779 ± 112b	678 ± 61c
	Shannon Index	9.34 ± 0.41a	8.77 ± 0.23b	7.64 ± 0.15c
	Simperson	0.002 ± 0.001c	0.003 ± 0.001b	0.005 ± 0.001a
Fungi	PD	112.91 ± 16.8b	132.72 ± 14.31a	96.18 ± 8.99c
	Pielou_eveness	0.78 ± 0.04a	0.71 ± 0.02a	0.64 ± 0.09b
	Chao1	408 ± 61a	447 ± 79a	332 ± 30b
	Shannon Index	5.94 ± 0.45a	6.28 ± 0.34a	5.36 ± 0.87b
	Simperson	0.034 ± 0.013b	0.044 ± 0.017b	0.099 ± 0.089a

Values are presented as mean (standard deviation). Values in the same columns followed by different letters differ significantly (p < 0.05, Duncan’s test).

### 3.2 The habitat distribution of ASVs within the same OTU

**Figure 4:**
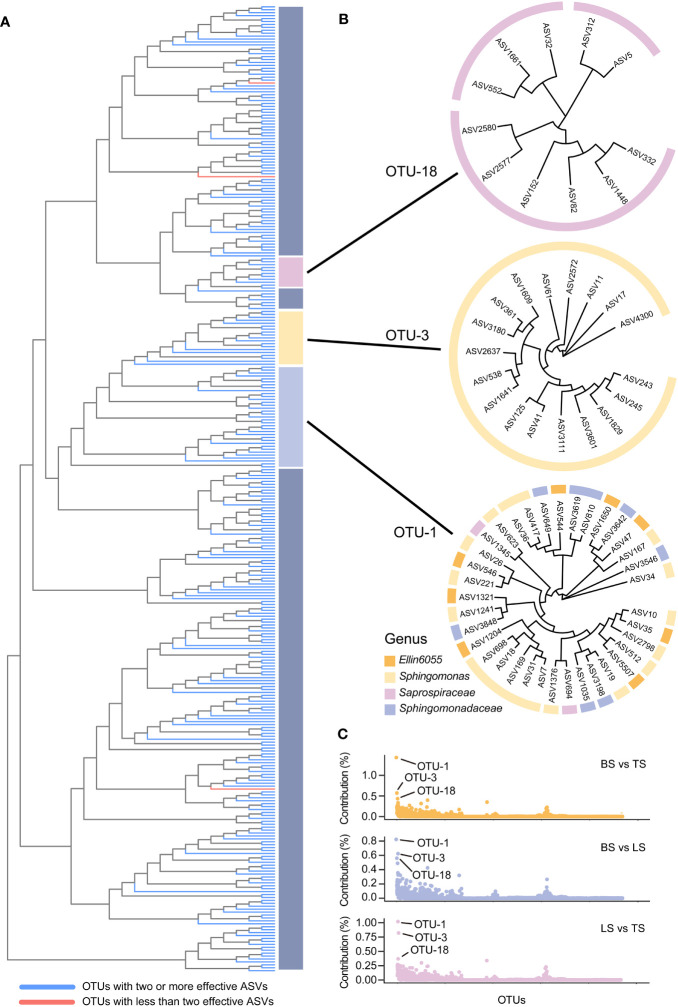
ASV-level microdiversity was prevalent within OTUs in bacteria **(A)**, OTU-1, OTU-3, and OTU-18 exhibited fine-scale diversity at ASV-level **(B)**, and the contribution of each OTUs in bacterial community **(C)**. SIMPER analysis was used to exhibit the contribution for each OTUs. Top 10% of relative abundance OTUs were used to exhibited the ASV-level microdiversity was prevalent within OTUs. OTU, operational taxonomic unit; ASV, amplicon sequence variants.

**Figure 5:**
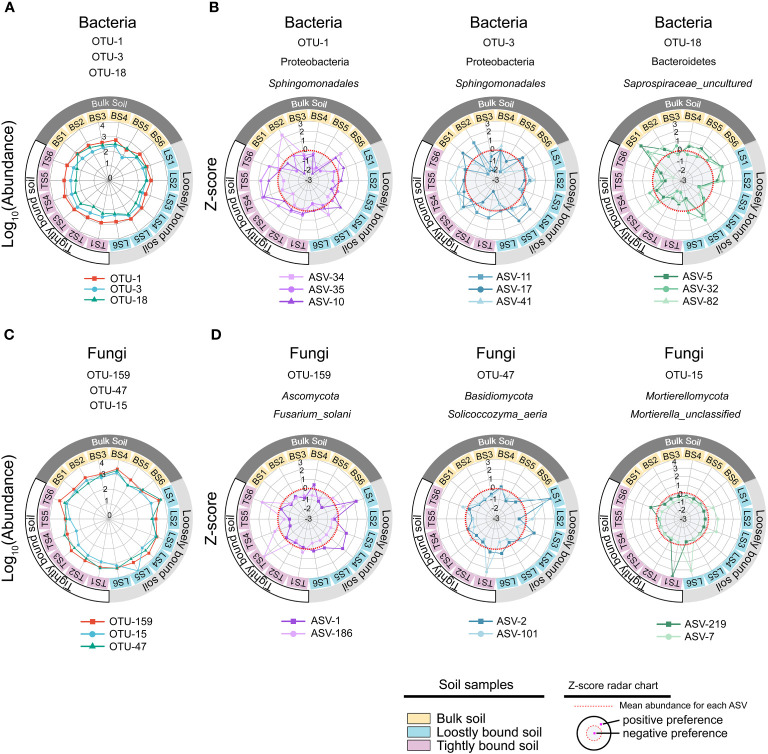
Different ecotypes of ASV from the same OTU show different habitat preference. The most prevalent species of bacteria **(A)** and fungi **(C)** in the dataset, and z-scores of the top three ASVs with the highest contribution rate in each OTUs **(B, D)**. The colours of the inner rings represent soil types. The z-scores indicate the habitat preference of each ASV. Each ASV was independently normalised though the z-score and could not be compared quantitatively among ASVs. The red dotted line indicates the mean abundance of each ASV, separating positive (white background), and negative (gray background) habitat preferences. OTU, operational taxonomic unit; ASV, amplicon sequence variants.

In this study, ASV-level microdiversity was prevalent within OTUs in bacteria and fungi, respectively (**Figures 4A**, **S1A**, **Tables S7**, **S8**). Among bacteria, SIMPER analysis showed that OTU-1, OTU-3, and OTU-18 had higher contributions across all OTUs among the bulk, loosely bound, and tightly bound soil samples (**Figure 4C**). OTU-1 (*Sphingomonadales*) had the highest abundance in the dataset, followed by OTU-3 (*Sphingomonadales*) and OTU-18 (*Saprospiraceae*) (**Figure S2A**). Therefore, we focused on OTU-1, OTU-3, and OTU-18 as potential agents to explore the ecological significance of microdiversity. Additionally, OTU-1, OTU-3, and OTU-18 exhibited fine-scale diversity at ASV-level (**Figure 4B**). Similar abundances across all soil compartments were observed for OTU-1, OTU-3, and OTU-18 (**Figure 5A**). The top three ASVs with the highest contribution rates within OTU-1, OTU-3, and OTU-18 exhibited different distributions and habitat preferences (**Figures 5B**, **S3**). For example, ASV-34 within OTU-1 showed a positive preference for the bulk and loosely bound soils, whereas ASV-35 showed a positive preference for tightly bound soil (**Figure 5B**). ASV-41 within OTU-3 showed a positive preference for loosely and tightly bound soils.

Among fungi, OTU-159, OTU-47, and OTU-15 exhibited fine-scale diversity at ASV-level (**Figure S1B**). Furthermore, SIMPER analysis showed that OTU-159, OTU-47, and OTU-15 had higher contributions across all OTUs among bulk, loosely bound, and tightly bound soil samples (**Figure S1C**). OTU-159 (*Fusarium_solani*) had the highest abundance in the dataset, followed by OTU-47 (*Solicoccozyma_aeria*) and OTU-15 (*Mortierella_unclassified*) (**Figure S2B**). OTU-159, OTU-47, and OTU-15 showed similar abundance across all soil compartments (**Figure 5C**). Under OTU-15, ASV-7 and ASV-219 were selected, both of which had a high contribution (**Figure S4**) and exhibited different habitat preferences (**Figure 5D**). ASV-1 with OTU-159 showed a positive preference for bulk and loosely bound soils, whereas ASV-186 showed a positive preference for tightly bound soil (**Figure 5D**). Similar results were observed for the other OTUs. For instance, ASV-79 within OTU-3 (*Lasiosphaeriaceae_unclassified*) exhibited a positive preference for tightly bound soil, while ASV-108 and ASV-226 showed a positive preference for bulk and loosely bound soils (**Figure S5**). Overall, abundant OTUs contain at least two effective ASVs were prevalent in all soil compartments; however, the ASVs that constituted the OTUs showed varying habitat preferences at the ASV level.

### 3.3 The abundance of constituent ASVs of the most persistence OTU was associated with environmental factors

**Figure 6:**
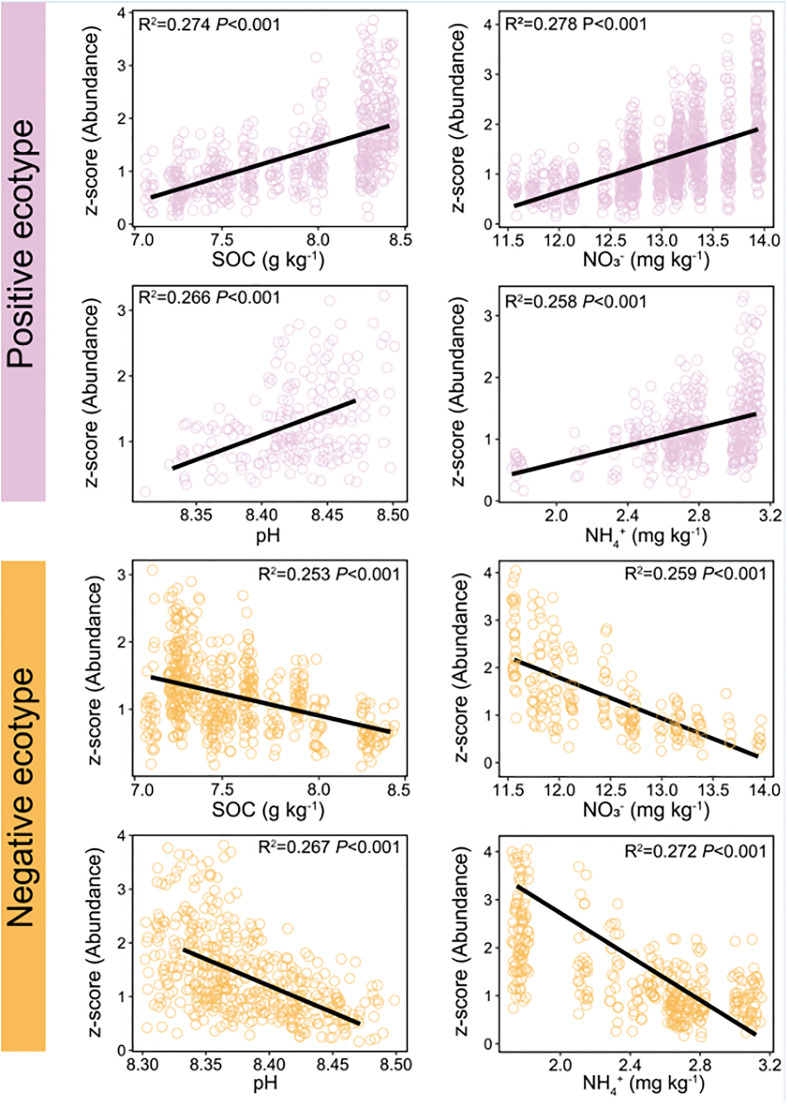
Relationship between soil environmental variables and the relative abundance of different ecotypes within the most persistence bacterial OTU (persistence > 0.75, variability < 2). Red line: the abundance of positive ecotypes increased with increasing levels of soil environmental variables. Blue line: the abundance of negative ecotypes decreased with increasing levels of soil environmental variables. OTU, operational taxonomic unit. Variability was measured as the coefficient of variation. Persistence was defined as the proportion of samples containing each lineage.

**Figure 7:**
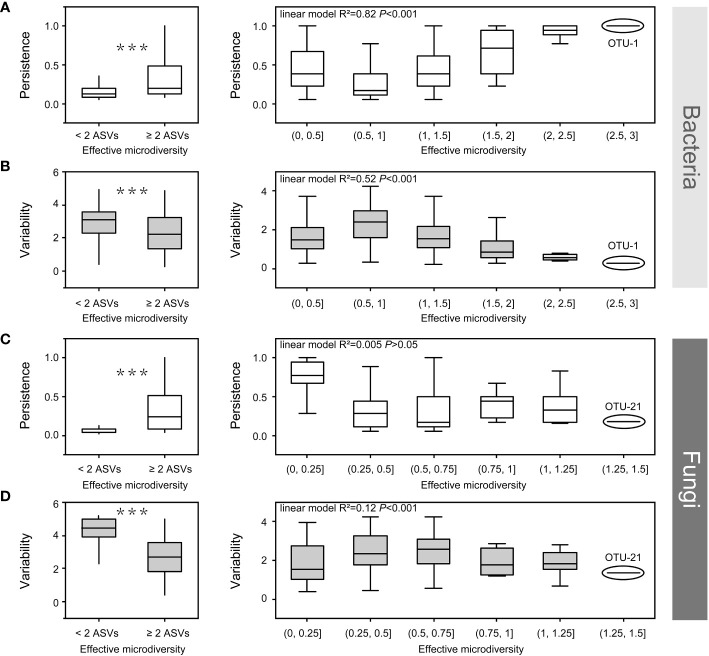
The effect of bacterial (a, b) and fungal microdiversity (c, d) on OTU persistence and variability. Boxplots show the effect of microdiversity (left: at least two ASVs vs. only one ASV, Wilcoxon test, ***p < 0.001). right: the correlation among persistence, variability and microdiversity. OTU, operational taxonomic unit; ASV, amplicon sequence variants.

The constituent ASVs of the most stable OTU in bacteria (persistence > 0.75, variability < 2) were selected to examine their association with environmental factors. The results showed that the abundance of the ASVs was significantly correlated with environmental factors, including SOC, pH, NO_3_^−^–N, and NH_4_^+^–N (**Figure 6**). Two ecotypes of ASVs were identified in this study: positive and negative. The relative abundance of positive ecotypes was positively correlated with the environmental factors, increasing with increase in the level of each environmental factor. In contrast, the relative abundance of negative ecotypes decreased with increasing levels of each environmental factor (**Figure 6**). Furthermore, we focused on OTU-1(*Sphingomonadales*) had the highest abundance in the dataset (**Figure S2A**) and richest microdiversity (**Figures 7A, B**), as a representative of the bacterial community. The abundance of ASVs (ASV-34 and ASV-35) with the highest contribution rate within OTU-1 showed different correlations with the environmental factors (**Figure S6**). Among fungi, OTU-21 had the richest microdiversity, but was not stable (OTU-21, persistence < 0.75, **Figure 7C**). Overall, in bacteria, the abundance of constituent ASVs of the most stable OTU was significantly correlated with environmental factors, indicating that microdiversification in response to environmental gradients from root surface to bulk soil is a general phenomenon.

### 3.4 Persistence and variability

Both bacteria and fungi, which are prevalent in all soil compartments, contain at least two ASVs. Some ASVs exhibited different habitat preferences, even from the same OTU. This indicates that ASVs from the same OTU had slightly different environmental preferences owing to the co-existence of different ecotypes. Therefore, we investigated the impact of microdiversity on the persistence and variability within bacterial species. The microdiversity of OTUs was calculated based on the abundance of their constituent ASVs. Regardless of the presence of either fungi or bacteria, OTUs containing at least two effective ASVs were more persistent and less variable than OTUs with single ASVs (Wilcoxon test, *p* < 0.001, **Figure 7**). In the bacterial community, variability decreased, and persistence increased with increasing microdiversity (**Figures 7A, B**). However, in fungal communities, variability and persistence were not significantly correlated with microdiversity (**Figures 7C, D**).

## 4 Discussion

### 4.1 Soil microbial community structure in the root-associated compartments

The results of the present study showed that α-diversity (Shannon and Chao1) was significantly lower in tightly bound soil than the loosely bound or bulk soils (**Table 1**), which was consistent with previous findings that diversity decreased from the bulk soil to the roots (Poole, 2017; Kuzyakov and Razavi, 2019; Semenov et al., 2019). Additionally, α-diversity was significantly correlated with environmental variables (**Tables 1**, **S5, S6**). Specific microorganisms are commonly selected by plant roots to colonize the rhizosphere, which can attract beneficial microorganisms to improve nutrient acquisition and combat pathogenic taxa (Dennis et al., 2010; Berendsen et al., 2012; Fan et al., 2017). The decrease in α-diversity from the bulk soil to roots could be attributed to root “filtration effect” (Dibbern et al., 2014; Fan et al., 2017). Generally, the rhizosphere is a highly selective environment that can select microbiome through two distinct processes (Fan et al., 2017; Fan et al., 2018). The first process involves the general recruitment of microbes to the proximity of the root, whereas the second process involves the transition of microbes from external to internal occupancy in the root (Edwards et al., 2015). Molecular signals from plants, including components of root exudates and possibly cell wall or membrane proteins, are involved in the selection of microbial community (Edwards et al., 2015; Kuzyakov and Razavi, 2019).

Additionally, the results of the present study showed that Proteobacteria and Bacteroidetes had higher relative abundance in tightly bound soil (**Figure 1A**, **Table S2**), which was consistent with previous findings in maize rhizosphere (Aira et al., 2010; Chauhan et al., 2011; Peiffer et al., 2013; Philippot et al., 2013; Huang et al., 2022). Proteobacteria and Bacteroidetes are considered r-strategists that respond to labile carbon sources or fast-growing microbiota with population opportunity fluctuations (Fierer et al., 2007; Peiffer et al., 2013). Generally, r-strategists have the ability to utilize a broad range of root-derived carbon substrates (Philippot et al., 2013). In contrast, we observed that Acidobacteria, Chloroflexi, and Verrucomicrobia were more abundant in loosely bound and bulk soils (**Figure 1A**, **Table S2**). Acidobacteria, Chloroflexi, and Verrucomicrobia have previously been described as K-strategists (Fierer et al., 2007). K-strategists or slower growing microbiota are generally considered to be enriched in bulk soil (Peiffer et al., 2013). Furthermore, the results of the present study showed that Ascomycota had a higher relative abundance in tightly bound soil than in the loosely bound and bulk soils. In contrast, the loosely bound and bulk soils had a higher abundance of Basidiomycota (**Figure 1B**, **Table S3**), which was consistent with previous reports in maize (Philippot et al., 2013; Fadiji et al., 2021). Basidiomycota has been previously described as a K-strategist (Yelle et al., 2008; Bastian et al., 2009), which could be responsible for the increase in its abundance in loosely bound and bulk soils. Overall, these results suggest that the soil compartments exhibited distinct microbiota structures and revealed variations in the abundance of bacteria (i.e., Proteobacteria, Bacteroidetes, Acidobacteria, Chloroflexi, and Verrucomicrobia) as well as fungi (i.e., Ascomycota and Basidiomycota) phyla.

### 4.2 ASVs reveal fine-scale diversity within the same OTU

The results of the present study showed that OTU and ASV resolutions were consistent in explaining the bacterial and fungal community structures of the samples (**Figures 2**, **3**). Recently, taxon has been defined based on the exact nucleotide sequences (also termed amplicon sequence variants) of marker genes (Callahan et al., 2017). Previously, a large field study was conducted to evaluate whether OTUs and ASVs offer different ecological outcomes. Glassman and Martiny (2018) reported that OTU and ASV methods exhibit similar ecological results, and that ASV only increases the genetic resolution of bacterial and fungal diversity, which was confirmed by the results of the present study. Specifically, this result suggests that our taxonomic resolution was sufficient to yield ecologically meaningful results.

In this study, the top 3 of relative abundance OTUs in bacteria and fungi showed that different ecotypes of ASVs were present in a single OTU (**Figures 4B**, **S1B**). Similarly, previous studies have identified microdiversity in soil microbiota in the rhizosphere of maize during different growth stages and root locations (i.e., rhizosphere and rhizoplane) (Di Cello et al., 1997; Seldin et al., 1998; Dalmastri et al., 1999; Schloter et al., 2000). Microdiversity in microbial communities could be attributed to several mechanisms (Larkin and Martiny, 2017), including horizontal gene transfer (Doolittle, 2012; Shapiro et al., 2012), single nucleotide polymorphisms (Kerr et al., 2002; Vellend, 2010; Hanson et al., 2012), and other genomic mutations (Tenaillon et al., 2012; O’Brien et al., 2013). The mutation frequency can be significantly augmented under certain environmental stress conditions, leading to episodic rapid evolution or macroevolution (Woese, 1987). Chromosomal rearrangements in bacterial species have the potential to drive diversification and give rise to phenotypes with distinct ecological niche occupancy abilities (Ginard et al., 1997; Dekkers et al., 1998). Gene conversions or duplications are outcomes of intragenomic recombination. Random recombination of gene loci and a net-like evolutionary process may result in the emergence of separate lineages (Smith et al., 1995; de la Cruz and Davies, 2000). Finally, DNA acquisition enables the acquisition of new capabilities in a single step, which could confer significant advantages for colonizing new habitats (Schloter et al., 2000). Moreover, events of horizontal gene transfer have the potential to initiate subsequent evolutionary processes and thus play a crucial role in generating microdiversity leading up to speciation (de la Cruz and Davies, 2000). For instance, it appears that non-toxigenic strains of *Bacteroides fragilis* have undergone evolution through horizontal gene transfer from diverse organisms (Franco et al., 1999). Notably, the microdiversity was influenced by the soil, maize cultivar, and root compartment, with the soil exerting a dominant influence (Dalmastri et al., 1999). This suggests that the microdiversity might be influenced by soil-related environmental factors.

Microorganisms rely on genetic diversity to adapt to the highly heterogeneous and fluctuating environment of the rhizosphere (Milkman, 1990; Whittam, 1992; Poole, 2017). Interestingly, the results of the present study showed that ASV-level microdiversity was prevalent within OTUs and was not merely within the top 3 of relative abundance OTUs in bacteria and fungi (**Figures 4A**, **S1A**, **Tables S7**, **S8**). Therefore, we concluded that ASV exhibited microdiversity in OTUs in root-associated soil compartments of mature maize plants growing under field conditions.

Further analysis of the constituent ASVs of the top 3 of relative abundance OTUs in bacteria and fungi showed that the ASVs exhibited different distributions and habitat preferences (**Figures 5B, D**), indicating that the ecotypes occupy different habitats. The co-existence of ecotypes formed by the splitting of microbial populations is often the result of different resource utilisation or the availability of specialised spatial or temporal niches (Good et al., 2018). Similarly, microbial populations have tens or hundreds of co-existing ecotypes that are formed via differentiation into distinct ecological niches to avoid competitive exclusion (Torsvik and Øvreås, 2002; Kashtan et al., 2014; Foster et al., 2017). Previous studies have reported that microdiversity is driven by a wide range of environmental factors, such as light, temperature, nutrients, and carbon substrates (Larkin and Martiny, 2017). In the rhizosphere, roots release various organic substrates to change the biogeochemistry of the environment (Wang et al., 2002). Variations in environmental factors in maize rhizosphere can affect intraspecific genetic diversity within a single species (Seldin et al., 1998; Dalmastri et al., 1999). For example, OTU-1 (*Sphingomonadales*) was prevalent in all soil compartments (**Figure 5A**). *Sphingomonadales* is associated with soil pH (Peiffer et al., 2013; Zhou et al., 2015; Wang et al., 2019), and is the dominant order in the rhizosphere of maize under higher soil pH (Zhang et al., 2021). However, the abundance of ASV-34 increased, and ASV-35 decreased with increasing soil pH, among ASVs in OTU-1 (**Figure S6**), with similar correlations observed for SOC, NO_3_^–^N, and NH_4_^+^–N (**Figure S6**). These results are in line with a previous report that fluctuating environmental factors in maize rhizosphere affect ecotypes within a single species (Latour et al., 1996; Dalmastri et al., 1999; Kandeler et al., 2002). Thereafter, we focused on the constituent ASVs of the most persistence OTUs in bacteria (persistence OTUs: persistence > 0.75, variability < 2) to reveal the potential relationship with soil properties. The results of the present study showed that the constituent ASVs of the most stable OTUs were mainly divided into positive and negative correlate with environmental factors (**Figure 6**), which is consistent with the fact that microdiversity is affected by environmental factors (Larkin and Martiny, 2017).

The results of the present study showed that ASV-level microdiversity was prevalent within the same OTU in root-associated soil compartments of mature maize plants growing under field conditions. In bacteria, the constituent ASVs of the most persistence OTUs were mainly divided into positive and negative correlate with environmental factors, indicating that environmental factors had a significant effect on microdiversity in the maize roots to bulk soil zone. Notably, other environmental variables not examined in this study, such as temperature, nutrient availability, and soil moisture, might also influence microdiversification in maize rhizosphere, resulting in closely related strains, each optimised for a particular ecological niche.

### 4.3 Microdiversity sustain species distribution along environmental gradients

In the present study, OTUs containing at least two ASVs showed higher persistence and lower variability in both the bacterial and fungal communities (**Figure 7**). In bacterial community, persistence increased with increasing microdiversity, whereas variability decreased with increasing microdiversity (**Figures 7A, B**). Rich biodiversity ensures the maintenance of bacterial populations, and distinct ecotypes within a species, sustaining species distribution along environmental gradients (Schloter et al., 2000; Shade et al., 2012; Konopka et al., 2015). In contrast, persistence and variability had no significant relationship with fungal microdiversity (**Figures 7C, D**), which could be attributed to the low microdiversity of the fungi community. For example, OTU-1 (bacterial OTU with the richest microdiversity) had a richer microdiversity than OTU-21(fungal OTU with the richest microdiversity) (**Figure 7**). Moreover, a previous study reported that environmental factors have a weaker effect on fungal community in compartments close to the roots (Zhang et al., 2017). Additionally, although variations in environmental factors may affect some species in the rhizosphere, these variations may have limited effects on strains or ecotypes within a single species in fungi, which could have contributed to the low microdiversity of the fungal community. Overall, the rich microdiversity sustain species distribution along environmental gradients from the root surface to the bulk soil in mature maize plants growing under field conditions.

## 5 Conclusion

In summary, our findings showed that ASVs exhibited microdiversity within OTUs in the root-associated soil compartments of mature maize plants growing under field conditions. Additionally, environmental factors in root-associated soil compartments of maize may drive the microdiversity in microbial community structure. Moreover, the rich microdiversity of bacterial community at the ASV-level may improve the stability of the microbial population in root-associated soil compartments of maize. In contrast, there was no correlation between microdiversity and the stability of fungal community, which was attributed to the low sensitivity of the fungal community to environmental factors in soil compartments close to the roots.

## Data availability statement

The datasets presented in this study can be found in online repositories. The names of the repository/repositories and accession number(s) can be found below: https://www.ncbi.nlm.nih.gov/, PRJNA997935.

## Author contributions

XF: Conceptualization, Data curation, Formal Analysis, Investigation, Methodology, Software, Writing – original draft. QF: Writing – review & editing, Data curation. XY: Writing – review & editing, Formal Analysis. HC: Supervision, Writing – review & editing. XZ: Data curation, Writing – review & editing. SL: Funding acquisition, Resources, Supervision, Writing – review & editing.
