# Supplementary material for: Microdiversity sustains the distribution of rhizosphere-associated bacterial species from the root surface to the bulk soil region in maize crop fields
Source: Front Plant Sci. 2023 Oct 12;14:1266218. doi: 10.3389/fpls.2023.1266218 (PMC10613529; doi:10.3389/fpls.2023.1266218)
Supplement: Supplementary file 1 [file DataSheet_1.docx]

# Supplementary information

| **Year** | **Samples** | **SOC** g kg^-1^ | **pH** | **NO_3_^－^–N** mg⋅kg^-1^ | **NH_4_^＋^–N** mg⋅kg^-1^ | **TN** g kg^-1^ |
| --- | --- | --- | --- | --- | --- | --- |
| **2019** | **TS** | 8.19±0.08a | 8.37±0.01a | 14.55±0.35a | 2.07±0.19b | 0.75±0.04a |
|  | **LS** | 7.51±0.33b | 8.38±0.03a | 12.44±0.67b | 2.79±0.18a | 0.75±0.02a |
|  | **BS** | 7.44±0.32b | 8.40±0.04a | 12.47±0.76b | 2.84±0.21a | 0.77±0.03a |
| **2020** | **TS** | 8.12±0.13a | 8.42±0.05a | 14.18±0.14a | 2.11±0.20b | 0.75±0.03a |
|  | **LS** | 7.65±0.25b | 8.39±0.03a | 12.50±0.65b | 2.81±0.08a | 0.77±0.04a |
|  | **BS** | 7.42±0.17b | 8.41±0.03a | 12.61±0.82b | 2.95±0.05a | 0.74±0.03a |
| **2021** | **TS** | 8.20±0.16a | 8.41±0.05a | 14.11±1.37a | 2.24±0.43b | 0.74±0.02a |
|  | **LS** | 7.50±0.22b | 8.40±0.04a | 12.97±0.38b | 2.84±0.23a | 0.74±0.04a |
|  | **BS** | 7.45±0.26b | 8.40±0.05a | 11.94±0.31b | 2.80±0.26a | 0.76±0.03a |

**Table S1** ANOVA test of soil physicochemical property under distinct soil compartments of maize rhizosphere

*Values are means ±SD. Values at the same columns followed by different letters differed significantly at *P*<0.05 ±Duncan’s test.

**Table S2** Comparison on relative abundance of bacterial taxa among different soil compartments.

| **Taxanomy±%** | **Compartments** | | |
| --- | --- | --- | --- |
|  | **BS** | **LS** | **TS** |
| Proteobacteria | 0.36±0.03b | 0.35±0.01b | 0.44±0.03a |
| Bacteroidetes | 0.12±0.03b | 0.12±0.04b | 0.15±0.06a |
| Acidobacteria | 0.14±0.02a | 0.13±0.02a | 0.10±0.01b |
| Gemmatimonadetes | 0.10±0.02ab | 0.11±0.01a | 0.07±0.02b |
| Actinobacteria | 0.07±0.02b | 0.10±0.01a | 0.11±0.02a |
| Chloroflexi | 0.06±0.01a | 0.06±0.02a | 0.04±0.01b |
| Nitrospirae | 0.03±0.01a | 0.02±0.01ab | 0.01±0.01b |
| Verrucomicrobia | 0.07±0.02a | 0.06±0.03a | 0.01±0.01b |
| othres | 0.04±0.02a | 0.03±0.01b | 0.04±0.02a |

*Values are means ±SD. Values at the same columns followed by different letters differed significantly at P<0.05 ±Duncan’s test.

**Table S3** Comparison on relative abundance of fungal taxa among different soil compartments.

| **Taxanomy±%** | **Compartments** | | |
| --- | --- | --- | --- |
|  | **BS** | **LS** | **TS** |
| Ascomycota | 0.71±0.03b | 0.70±0.03b | 0.82±0.02a |
| Basidiomycota | 0.17±0.05a | 0.15±0.07a | 0.11±0.03b |
| Mortierellomycota | 0.09±0.04ab | 0.14±0.07a | 0.04±0.01b |
| Mucoromycota | 0.03±0.02a | 0.01±0.01a | 0.02±0.01a |

*Values are means ±SD. Values at the same columns followed by different letters differed significantly at P<0.05 ±Duncan’s test.

**Table S4** ANOSIM, ADONIS test of separable compartments on microbial community Unweighted UniFrac distances matrix.

| **Microbes** | **Dissimilarity** | **ANOSIM** | | **ADONIS** | |
| --- | --- | --- | --- | --- | --- |
|  | **All** | **R** | **P** | **F** | **P** |
| **Bacteria** | **BS vs LS** | 0.52 | 0.004 | 1.91 | 0.002 |
|  | **BS vs TS** | 0.48 | 0.001 | 1.56 | 0.001 |
|  | **LS vs TS** | 0.44 | 0.002 | 2.03 | 0.001 |
| **Fungi** | **BS vs LS** | 0.35 | 0.037 | 1.26 | 0.017 |
|  | **BS vs TS** | 0.37 | 0.007 | 1.66 | 0.001 |
|  | **LS vs TS** | 0.21 | 0.002 | 1.37 | 0.002 |

**Table S5** The correlations were determined by Spearman Correlation between the alpha diversity and environmental variables in bacteria. ’**’ is p<0.01; ’*’ is p<0.05.

| **BS** | **pH** | **SOC** | **TN** | **NO_3_^－^–N** | **NH_4_^＋^–N** |
| --- | --- | --- | --- | --- | --- |
| **Pielou_eveness,** | -0.562 | 0.927** | 0.742 | 0.959** | 0.643 |
| **Chao1** | -0.743 | 0.9* | 0.906* | 0.844* | 0.944** |
| **Shannon Index** | -0.91* | 0.906* | 0.781 | 0.848* | 0.813* |
| **PD** | -0.786 | 0.88* | 0.735 | 0.838* | 0.937** |
| **Simperson** | -0.743 | 0.984** | 0.746 | 0.99** | 0.786 |
| **LS** | **pH** | **SOC** | **TN** | **NO_3_^－^–N** | **NH_4_^＋^–N** |
| **Pielou_eveness** | 0.629 | 0.933** | 0.579 | 0.91* | 0.88* |
| **Chao1** | 0.686 | 0.903* | 0.9* | 0.835* | 0.916* |
| **Shannon Index** | 0.779 | 0.862* | 0.496 | 0.666 | 0.873* |
| **PD** | 0.756 | 0.931** | 0.856* | 0.869* | 0.929** |
| **Simperson** | 0.778 | 0.96** | 0.623 | 0.834* | 0.968** |
| **TS** | **pH** | **SOC** | **TN** | **NO_3_^－^–N** | **NH_4_^＋^–N** |
| **Pielou_eveness** | -0.853* | 0.916* | -0.066 | 0.947** | 0.947** |
| **Chao1** | -0.871* | 0.89* | -0.16 | 0.883* | 0.889* |
| **Shannon Index** | -0.722 | 0.976** | -0.405 | 0.86* | 0.906* |
| **PD** | -0.677 | 0.756 | -0.24 | 0.771 | 0.712 |
| **Simperson** | -0.761 | 0.942** | -0.132 | 0.969** | 0.947** |

**Table S6** The correlations were determined by Spearman Correlation between the alpha diversity and environmental variables in fungi. ’**’ is p<0.01; ’*’ is p<0.05.

| **BS** | **pH** | **SOC** | **TN** | **NO_3_^－^–N** | **NH_4_^＋^–N** |
| --- | --- | --- | --- | --- | --- |
| **Pielou_eveness,** | -0.698 | 0.977** | 0.833* | 0.983** | 0.738 |
| **Chao1** | -0.647 | 0.97** | 0.815* | 0.985** | 0.726 |
| **Shannon Index** | -0.746 | 0.961** | 0.854* | 0.948** | 0.79 |
| **PD** | -0.818* | 0.959** | 0.777 | 0.927** | 0.923** |
| **Simperson** | 0.692 | -0.941** | -0.73 | -0.953** | -0.737 |
| **LS** | **pH** | **SOC** | **TN** | **NO_3_^－^–N** | **NH_4_^＋^–N** |
| **Pielou_eveness** | 0.853* | 0.994** | 0.657 | 0.942** | 0.932** |
| **Chao1** | 0.679 | 0.967** | 0.666 | 0.887* | 0.988** |
| **Shannon Index** | 0.735 | 0.943** | 0.647 | 0.975** | 0.845* |
| **PD** | 0.613 | 0.901* | 0.852* | 0.865* | 0.944** |
| **Simperson** | -0.527 | -0.91* | -0.689 | -0.835* | -0.961** |
| **TS** | **pH** | **SOC** | **TN** | **NO_3_^－^–N** | **NH_4_^＋^–N** |
| **Pielou_eveness** | -0.722 | 0.949** | -0.396 | 0.844* | 0.88* |
| **Chao1** | -0.672 | 0.737 | -0.312 | 0.704 | 0.673 |
| **Shannon Index** | -0.733 | 0.94** | -0.429 | 0.82* | 0.859* |
| **PD** | -0.727 | 0.874* | -0.298 | 0.837* | 0.826* |
| **Simperson** | 0.741 | -0.892* | 0.374 | -0.801 | -0.826* |

Fig.S1


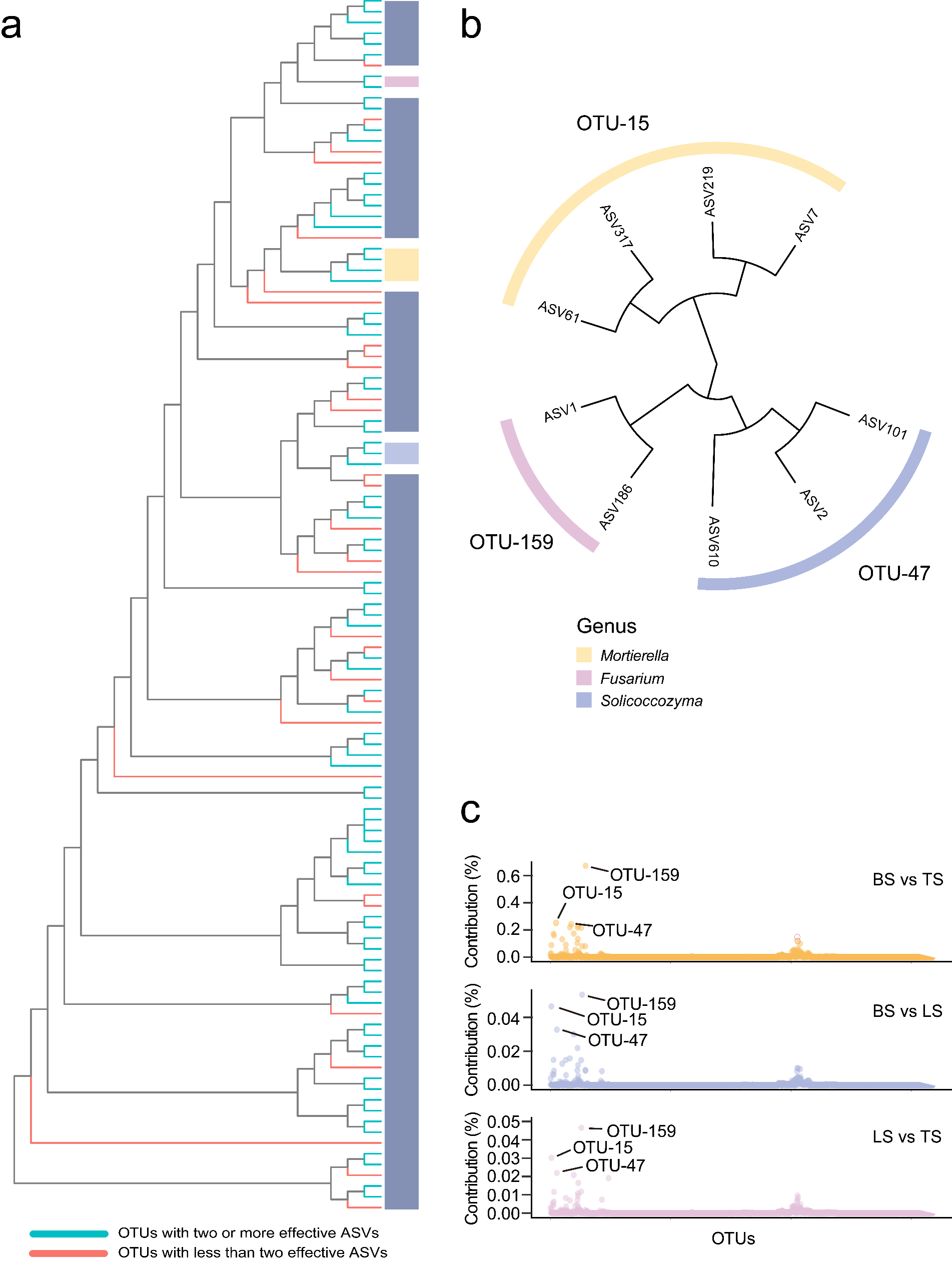


**Fig. S1.** ASV-level microdiversity was prevalent within OTUs in fungi (a), OTU-15, OTU-159, and OTU-47 exhibited fine-scale diversity at ASV-level (b), and the contribution of each OTUs in fungal community. SIMPER analysis was used to exhibit the contribution for each OTUs. Top 10% of relative abundance OTUs were used to exhibited the ASV-level microdiversity was prevalent within OTUs.

Fig.S2


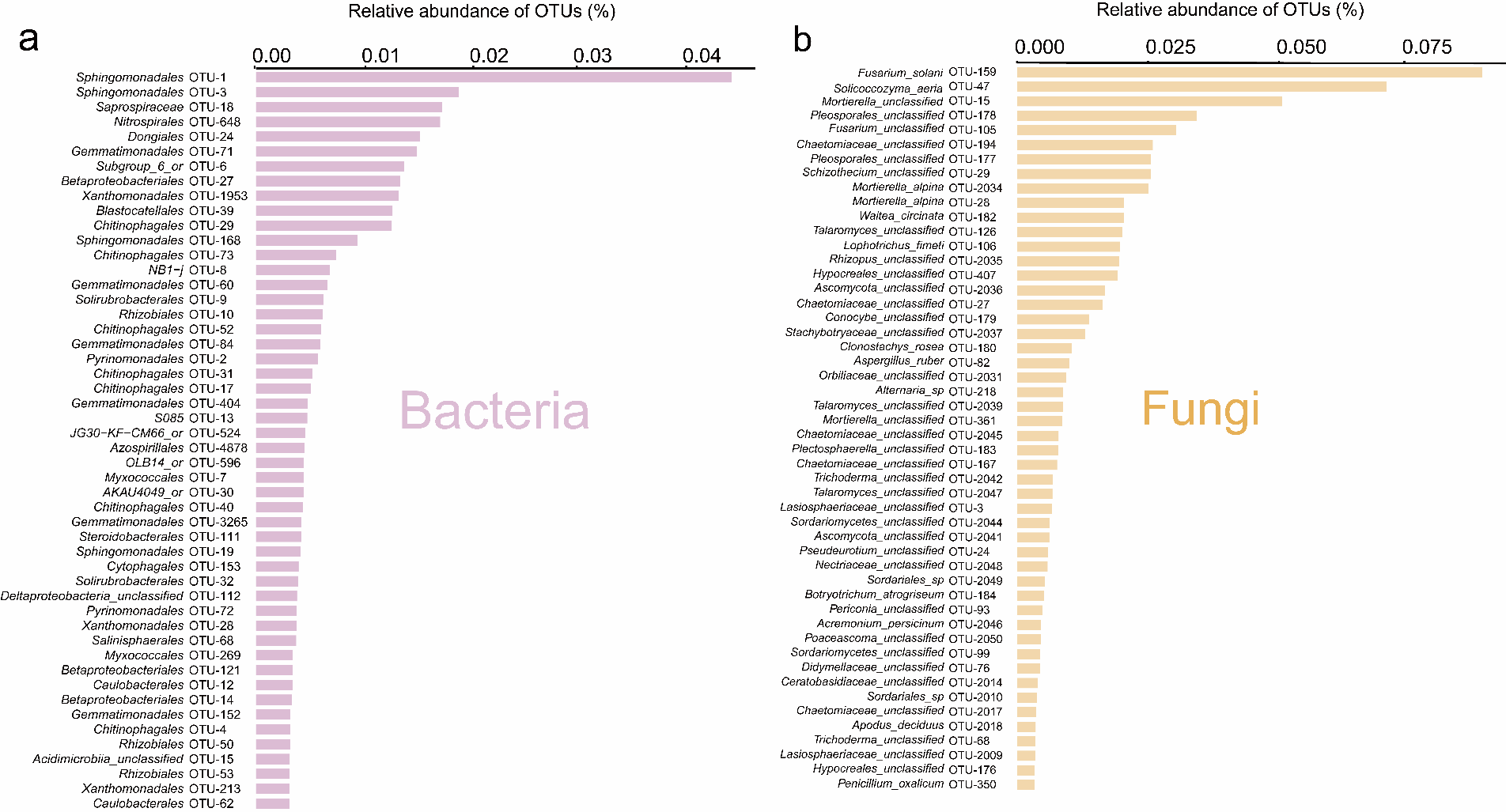


**Fig. S2.** Top 50 of relative abundance OTUs in bacteria and fungi, respectively.

Fig.S3


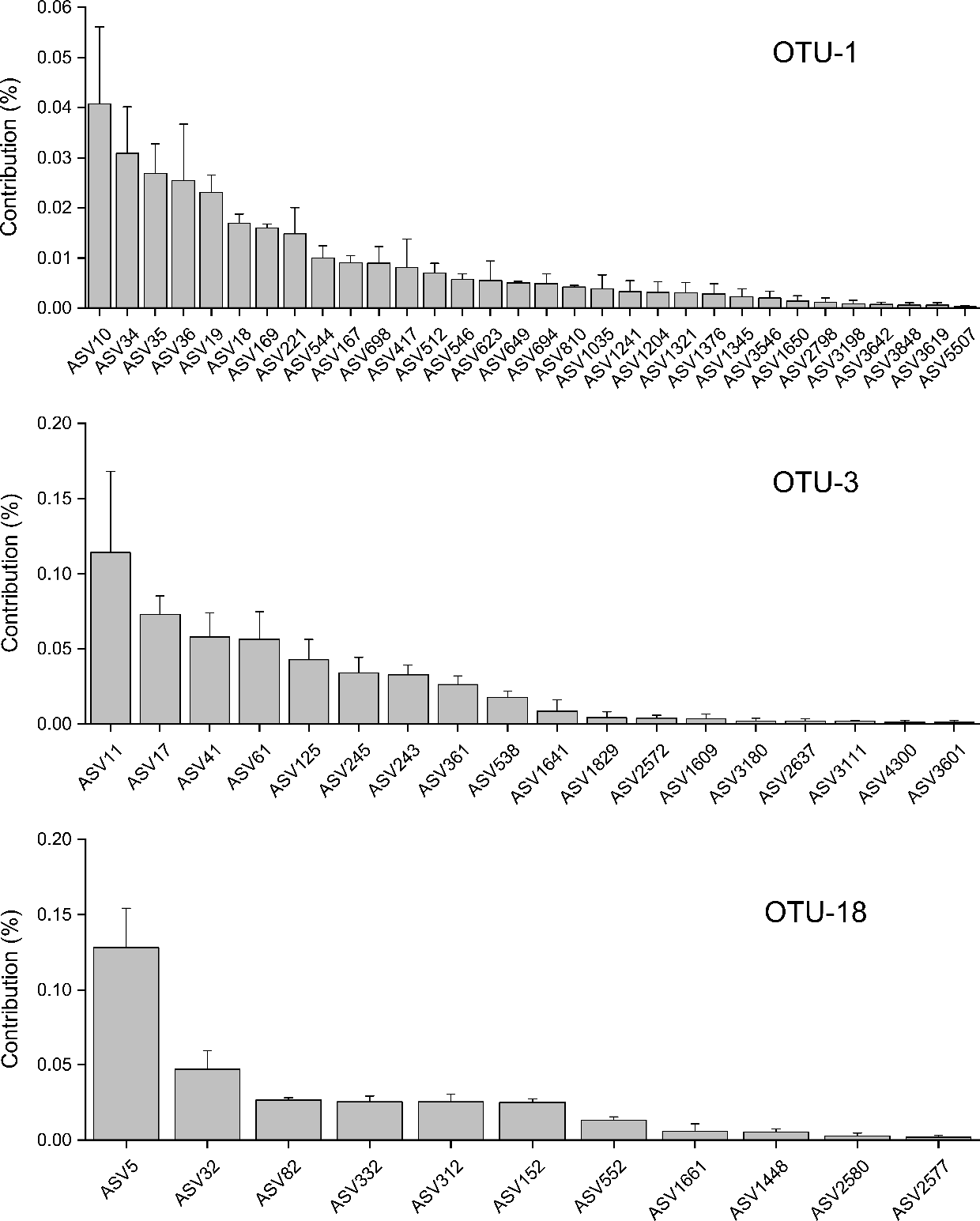


**Fig. S3.** The contribution of each ASVs contained in the same OTU in bacterial community. SIMPER analysis was used to exhibit the contribution for each ASVs.

Fig.S4


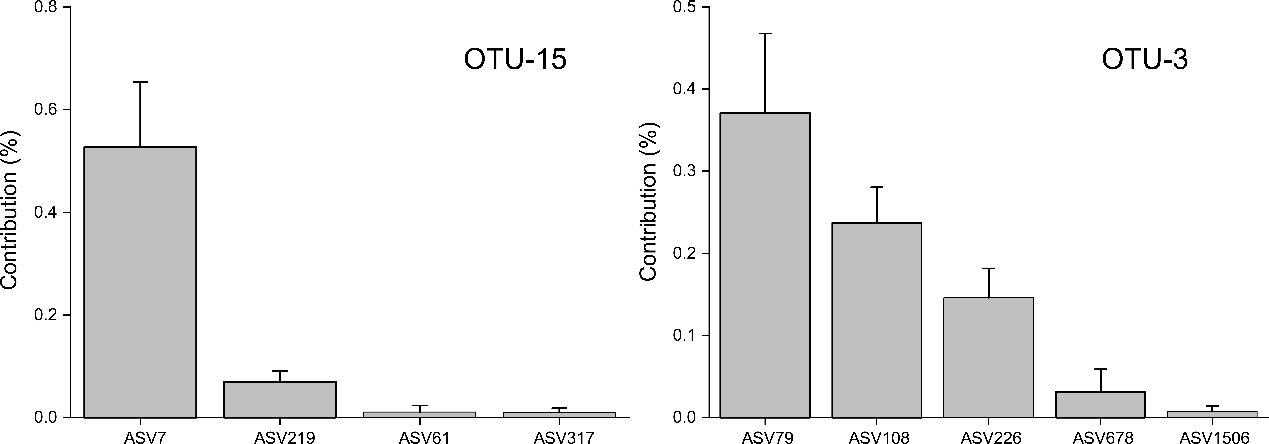


**Fig. S4.** The contribution of each ASVs contained in the same OTU in fungal community. SIMPER analysis was used to exhibit the contribution for each ASVs.

Fig.S5


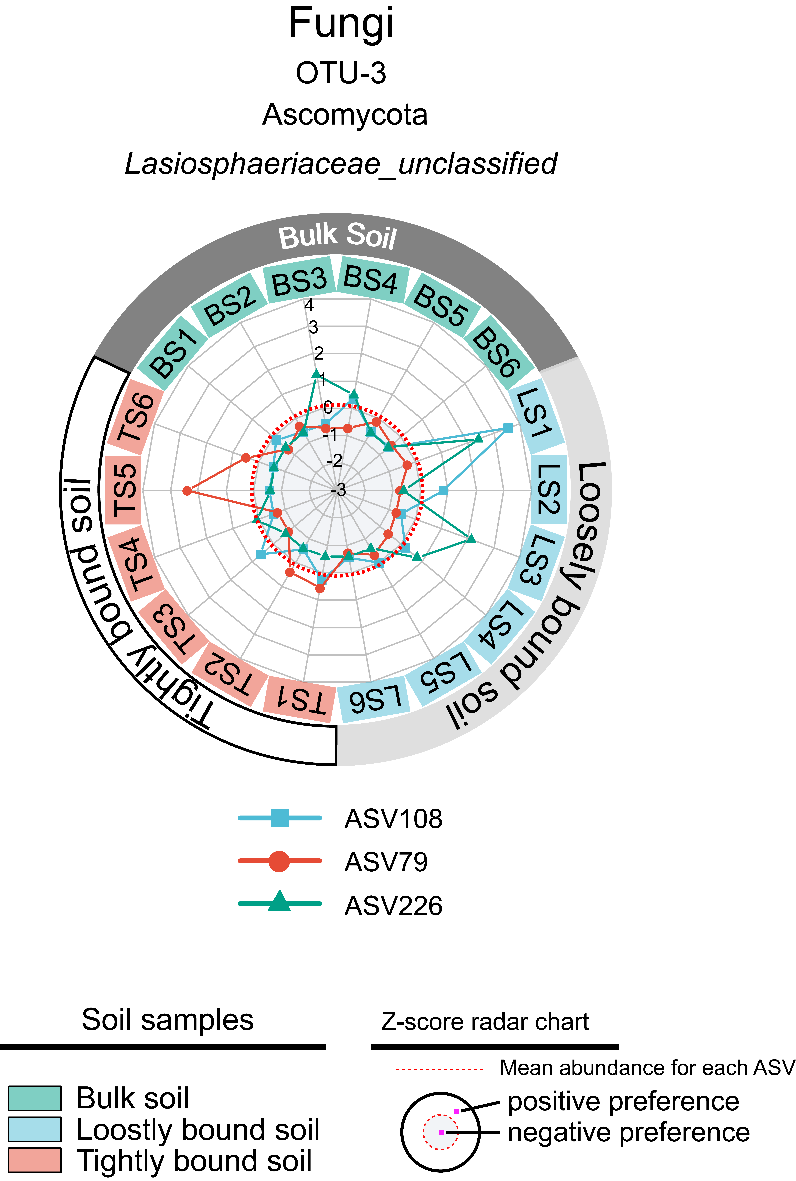


**Fig. S5.** Different ecotypes of ASV from the OTU-3 show different habitat preference. The colors of the inner-ring represent the soil types. z-scores indicate the habitat preference of each ASV. Each ASV was independently normalized though z-score, and cannot be compared quantitatively among ASVs. The red dotted line indicates the mean abundance of each ASV, separating positive ±white background and negative ±gray background habitat preferences.

Fig.S6


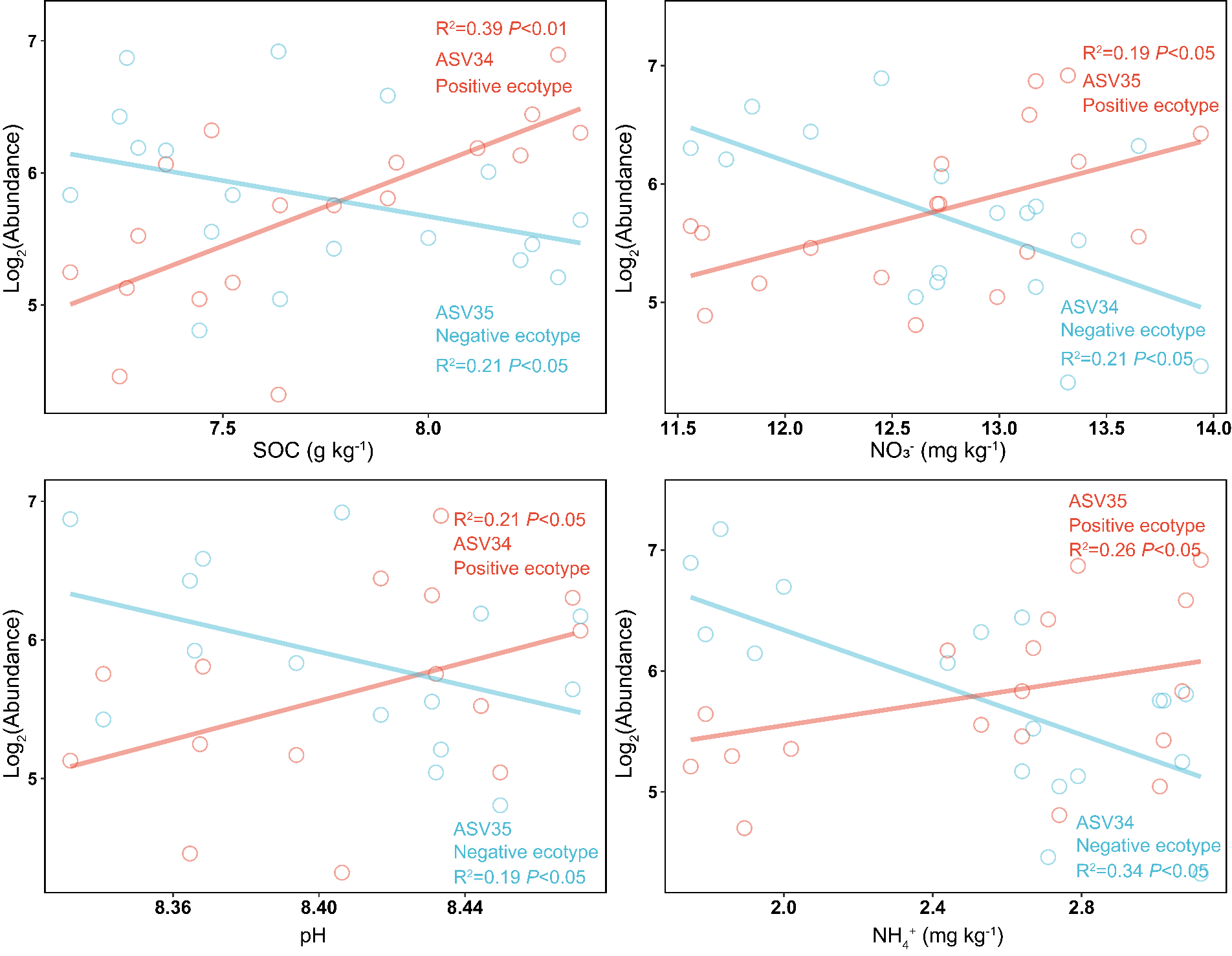


**Fig. S6.** Different ecotypes of ASV from the OTU-1 show different habitat preference.


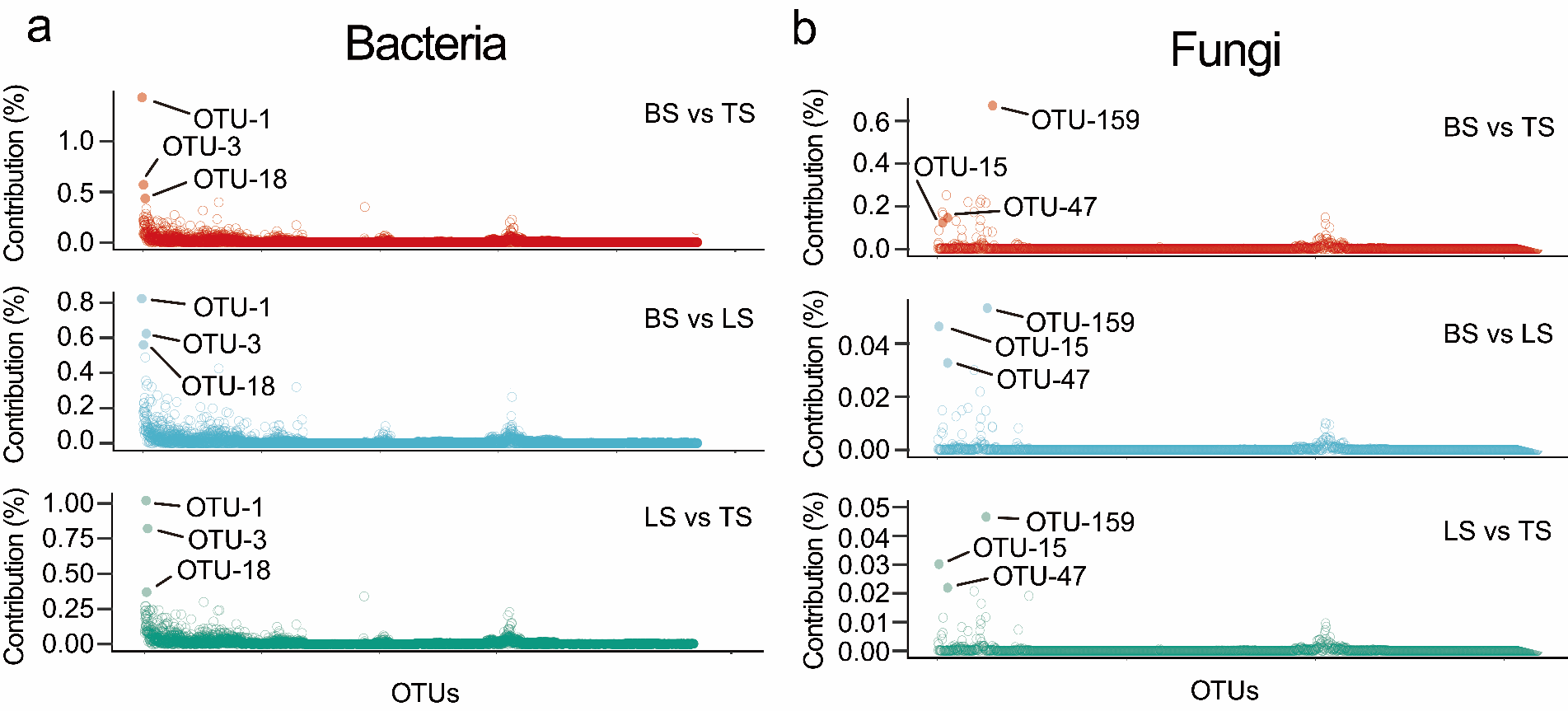


**Fig. S1.** The contribution of each OTUs contained in bacterial and fungal community. SIMPER analysis was used to exhibit the contribution for each OTUs.
